# Put your phone down! Perceived phubbing, life satisfaction, and psychological distress: the mediating role of loneliness

**DOI:** 10.1186/s40359-023-01359-0

**Published:** 2023-10-12

**Authors:** Alexandra Maftei, Cornelia Măirean

**Affiliations:** 1 Faculty of Psychology and Education Sciences, Department of Education Sciences, Alexandru Ioan Cuza University, Iasi, Romania; 2 Faculty of Psychology and Education Sciences, Department of Psychology, Alexandru Ioan Cuza University, 3 Toma Cozma Street, Iasi, Romania

**Keywords:** Phubbing, Life satisfaction, Psychological distress, Loneliness

## Abstract

The recent literature highlights the need to explore phubbing, a worrying growing phenomenon, and the potential associated factors and outcomes. Thus, the present study focused on perceived phubbing and the potential associations with life satisfaction and psychological distress. Additionally, this study examined the mediating role of loneliness. The cross-sectional, web-based study involved 720 Romanian adults aged 18 to 77 (*M* = 24.12, *SD* = 8.80, 74% females) who completed self-reported scales measuring perceived phubbing, and life satisfaction. Results suggested positive associations between perceived phubbing, loneliness, and psychological distress. A significant negative association was also found between loneliness and life satisfaction. Further analyses suggested that loneliness partially mediated the link between perceived phubbing and life satisfaction and between perceived phubbing and psychological distress. The study highlights the significant role of perceived phubbing when discussing psychological distress and life satisfaction, underlining the need to address further the (mis)use of digital devices (e.g., smartphones) within interpersonal relationships, as well as the need to understand better the outcomes of such behaviors to shape effective interventions in this regard.

**Supplementary Information:**

The online version contains supplementary material available at 10.1186/s40359-023-01359-0.

## Introduction

The explosion of communication technologies and digital devices within the past decade, especially smartphones, significantly changed how we connect and interact. According to a recent report, as of 2021, there were approximately 5.3 billion smartphone users worldwide [1]. The challenges and risks of the digitalized world we live in are growing [2], along with the benefits of the Internet [3], shaping a complex picture of the psychological implications of digital behaviors. Furthermore, smartphone usage can alter communication and social relationship routines and may cause unusual behavior. For instance, excessive smartphone use may reduce eye contact and limit connection and interaction, significantly affecting interpersonal relations [4]. This is all the more important since almost 90% of young users prefer messaging instead of face-to-face communication [5].

One of these challenges is *phubbing*, an increasingly worrying phenomenon [6], describing “the action of ignoring someone or multiple people during social events and using smartphones to check or use Facebook, WhatsApp or other social media applications” [7]. In other words, phubbing refers to “an individual giving more attention to their mobile phone when in face-to-face communication with another individual” [8]. The phubbing agent is called *phubber*, while the person experiencing phubbing (i.e., the one ignored in favor of the smartphone) is called *phubbee* [9]. Previous research shows that phubbing experiences are common, regardless of age [9]. However, studies also highlighted that older adults might consider phubbing behavior as particularly disruptive within family contexts when conversational expectations are violated and might contribute to negative emotional states [10, 11]. In the present study, the primary focus was on *phubbees*, i.e., people who experience phubbing.

## Phubbing, life satisfaction, and psychological distress

Though a novel research concept, due to its antisocial nature [12], phubbing has been the subject of an increasing number of studies examining its adverse outcomes on one’s social communication patterns and interpersonal relations [13]. Previous studies suggested that phubbing may be significantly and negatively linked to life satisfaction [14], though these results are based on phubbing experiences as an agent of phubbing, not the person who gets phubbed. Roberts and David [15] suggested that partner phubbing had a significant, direct negative impact on relationship satisfaction and an indirect impact on life satisfaction and depression. Furthermore, similar studies examining the impact of phubbing within relationships suggested that, among the most frequent outcomes, we might find increased jealousy, depressive symptoms, and lower relationship satisfaction [16, 17].

Other studies suggested that *phubbees* might experience a sense of devaluation and disrespect by the phubbing agent, in addition to a decrease in communication quality [17, 18]. Also, phubbing experiences negatively impact conversation quality and feelings of connectedness [19, 20]. Furthermore, in the extensive overview of phubbing, Al-Saggaf [21] describes the scarce, but growing evidence of the predictive role of psychological distress (e.g., anxiety, stress, and depressive symptoms) regarding phubbing behaviors [22, 23], as well as a potential consequence of being phubbed [24, 25] or partial and serial mediators between phubbing and maladaptive technology-related behaviors, such as smartphone addiction [25, 26]. Also, some studies suggested that phubbing might not have a significant, direct relationship with life satisfaction but an indirect relation mediated by other factors, such as relationship style [27].

Given these few results regarding the complex relationship between phubbing experiences, life satisfaction, and psychological distress, there is a need for further related studies to better understand the dynamics of these relations, especially concerning *phubbees*, an understudied group in the context of phubbing behaviors, especially among adults.

## The mediating role of loneliness

Loneliness is generally described as a negative emotional state that individuals experience when confronted with a discrepancy between their desired interpersonal relationships and those perceived as having [28], a sense of isolation or a state of absence from the community, family, or peers [29]. Loneliness is a commonly studied factor when discussing problematic Internet use (PIU), presenting small to medium associations with PIU in most cross-sectional studies [30]. Also, various studies highlighted the negative association between loneliness and life satisfaction [31] and the positive association between loneliness and psychological distress [32]. However, the literature gets significantly less generous when examining the link between phubbing experiences, a particular form of maladaptive Internet and technology use, and loneliness, and the emerging results are quite complex.

For example, Ergün et al. [24] suggested that phubbing (i.e., agent behavior) was negatively linked to loneliness and positively to life satisfaction. Phubbing seems connected to loneliness and social isolation [20], which can further exacerbate negative emotions and psychological distress [33]. The literature linking phubbing experiences (as a “receiver”, not an agent) and loneliness is scarce, but some previous data argued on the predictive role of peer rejection - which is also connected to phubbing [34] - on loneliness [35, 36]. Other studies suggested that loneliness mediates the link between phubbing experiences and social media addiction [37], which is significantly associated with high psychological distress [38] and low life satisfaction [39]. Xu and their collaborators [37] based their assumptions on the fact that phubbing experiences make participants feel rejected and neglected, increasing their loneliness. Also, Chotpitayasunondh and Douglas [18] suggested that increased phubbing negatively impacted communication quality perceptions and relationship satisfaction, and feelings of belonging (the opposite of loneliness) mediated these effects.

Finally, Ivanova et al. [40] suggested that higher loneliness might increase the mediating effect of phubbing, which further translates into depression among male participants. In contrast, in female participants, the authors reported that the examined mediation effect weakens as loneliness grows (phubbing correlates less strongly with depressive symptoms).

## Age, gender, relationship status, and time spent online

Previous studies suggested that gender might moderate the link between phubbing experiences (i.e., phubbing target) and how people might perceive phubbing as normative [9], with a stronger effect in male participants, compared to the same relationship in women. However, other studies suggested that females might be more prone to experience more loneliness [41, 42] compared to males, which, in turn, might make them more vulnerable regarding phubbing experiences [37]. Thus, males might hold a more normative view of phubbing, while females might experience more phubbing-related harm.

Regarding age, some previous studies suggested that older individuals generally consider phubbing behavior offensive and, similar to college-aged participants, consider it a violation of common courtesy, especially in one-on-one interactions [10, 11]. Also, since younger individuals tend to use the Internet, social media, and smartphones, in general, more than older people [43], one can assume that youth are more prone to experience phubbing. Thus, age might be a significant related factor in this regard.

Relationship status (e.g., single, romantic, or married) is also important when discussing loneliness, life satisfaction, or psychological distress. For example, Adamczyk and Segrin [44] suggested that relationship status might have a significant indirect effect on life satisfaction through loneliness and perceived social support. Similarly, Bucher et al. [31] suggested that committed relationships might increase life satisfaction and reduce loneliness. However, these effects might also be moderated by age [45]. Furthermore, married people seem to report higher well-being compared to those involved in a romantic relationship (without being married), or people who are not involved in a relationship at all [46]. Nevertheless, these associations are also subject to relationship quality in both female and male individuals [47].

Furthermore, previous studies also highlighted the significant relationships between time spent online, loneliness, life satisfaction, and psychological distress. For example, Stepanikova et al. [48] suggested that time spent on the Internet might be positively related to loneliness and negatively related to life satisfaction. Similar findings were reported by a growing number of studies, regardless of participants’ age and perceived social support [49].

## The present study

**Fig. 1:**
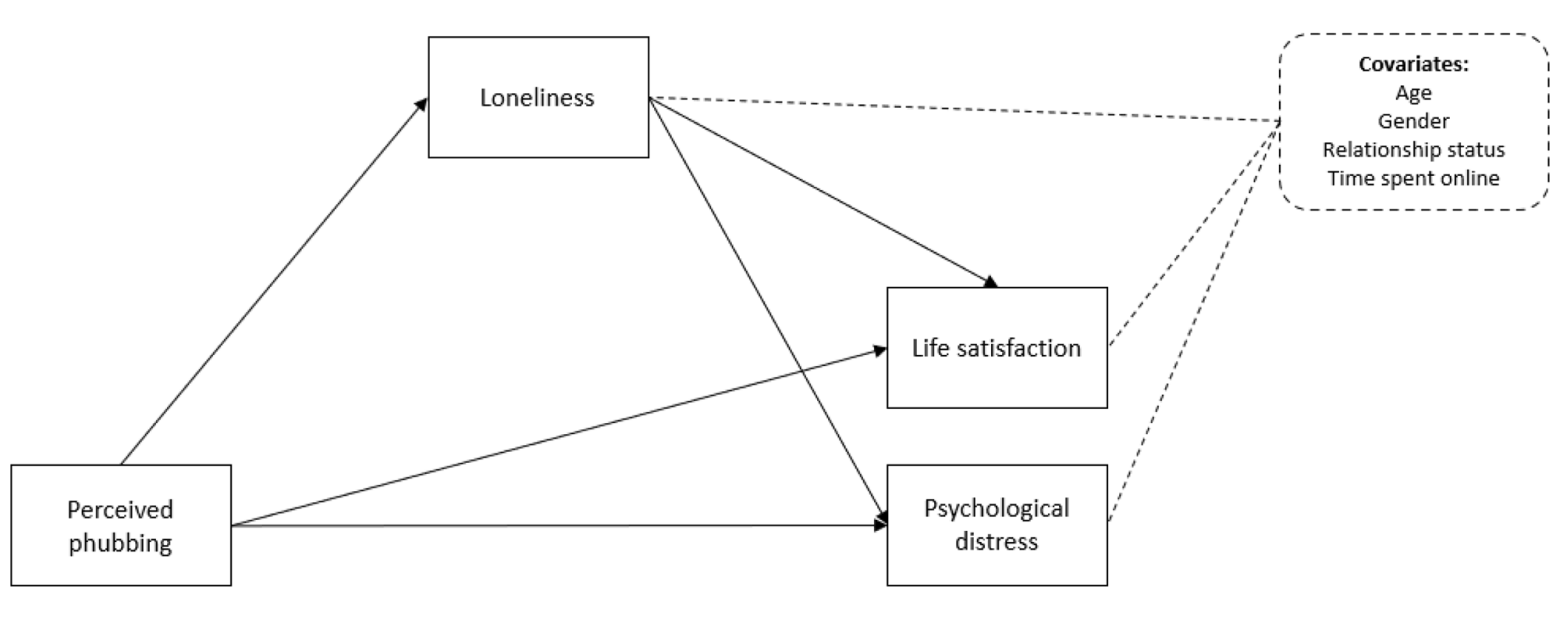
The proposed research model

Given the documented negative outcomes related to phubbing, as well as the need to further explore the risk factors and possible mental health outcomes in this regard, the present study aimed to investigate the link between perceived phubbing, life satisfaction, and psychological distress, and the mediating role of loneliness in this regard. The novelty of this study lies in the fact that (1) the study examined the link between phubbing experiences, loneliness, life satisfaction, and psychological distress, focusing on *phubbees*, and not on agents of phubbing, as most previous studies have; (2) the sample was formed by adults (phubbees), a rather understudied population regarding phubbing since most studies focused on teenagers [50, 51]. Moreover, though some studies examined phubbing among young adults [24], to our knowledge such studies are missing within the Romanian cultural space. The proposed research model (see Fig. 1) was primarily based on the stress and coping framework [52], which generally states that the extent to which a stressful event or situation affects one’s well-being/psychological distress depends on the context in which the stressor occurs [53]. Phubbing experiences could be perceived as stressors, and individuals’ responses to stress (such as feeling lonely) may impact their psychological well-being [54]. Furthermore, the subjective influence of a stressor is subject to one’s available coping resources [53], and social support (which is significantly and negatively related to loneliness; [55]) is commonly seen as one of the primary related resources due to its comprehensive nature [56].

Thus, the primary research questions (RQ) were the following: *RQ1*. Is there a significant link between phubbing, life satisfaction, and psychological distress?; *RQ2*. Does loneliness mediate this link, regardless of demographic characteristics (i.e., gender and age), relationship status, and the time spent online? Translating these questions into research assumptions, the hypotheses were the following:

(H1) Perceived phubbing will be positively related to psychological distress and negatively related to life satisfaction;

(H2) Loneliness would mediate the link between perceived phubbing, psychological distress, and life satisfaction when controlling for demographic and relational factors and the time spent online.

## Method

### Participants and procedure

The initial sample comprised 730 participants. Out of these 730, ten were excluded since they did not fit the age requirements, which was the only inclusion criterion (i.e., > 18 years). The final sample comprised 720 adults aged 18 to 77, *M*_age_ = 24.12, *SD* = 8.80, 74% (*N* = 533) females. Most participants were involved in a romantic relationship (44%), followed by those who reported being single (36.1%), married (18.2%), divorced (1.4%), or widowed (0.3%). Convenience sampling was used (i.e., the snowball sampling technique), and data were collected in 2022 using an online form distributed through social media groups. A sensitivity power analysis was performed using G*Power 3.1 [57] to identify the minimum sample size needed for the research design that was used, i.e., examining the relationships between two independent variables in each mediation model (i.e., perceived phubbing and loneliness) and a dependent variable (i.e., psychological distress/ life satisfaction). For a medium effect f2 = 0.15, with an alpha = 0.05 and power = 0.95, the minimum sample required was 107. Also, the Monte Carlo post-hoc power analysis for indirect effects [58] indicated that the sample size used in the present research had sufficient power for the proposed analysis.

The research was conducted following the 2013 Helsinki Declaration ethical guidelines and those approved by the Ethics Committee from the universities with which the authors are affiliated (ethical approval 2138/24.10.2022). All participants gave their informed consent to take part in this research. They were informed that their participation was voluntary, they could withdraw from the study at any point, and their responses would remain anonymous and confidential. The average time required to answer all questions was 20 min.

### Measures

The forward-backward translation approach was used to translate some of the scales from English to Romanian [59]. The minor inconsistencies between the original and back-translated versions were ironed out, yielding the final versions of each scale.

#### Psychological distress

The Depression, Anxiety, and Stress Scale (DASS-21; [60]) – the Romanian version [61, 62] was used to measure participants’ depression, anxiety, and stress symptoms, i.e., psychological distress. Items were measured on a 4-point Likert scale ranging from 0 (it does not apply to me at all) to 3 (it applies to me a lot most of the time). Cronbach’s α in the present study was 0.95. Higher scores indicated higher psychological distress.

#### Life satisfaction

The Satisfaction with Life Scale developed by Diener et al. [63] was further used. The 5-item scale measures participants’ global cognitive judgments of life satisfaction on a Likert scale ranging from 1 (strongly agree) to 7 (strongly agree). Cronbach’s alpha in the present study was 0.88. Higher scores indicated higher life satisfaction.

#### Perceived phubbing

Phubbing Experiences were measured using the 22-item Generic Scale of Being Phubbed developed by Chotpitayasunondh et al. [18]. The scale comprises three factors, i.e., Perceived Norms (α = 0.92), Feeling Ignored (α = 0.95), and Interpersonal Conflict (α = 0.91). The exact instructions were: “Think about your social interactions on the whole (e.g., with friends, acquaintances, family, your partner) and the extent to which the following statements apply to you. In my face-to-face social interactions with others” [18]. An overall score of the scale was used, which had an internal consistency of 0.95. Higher scores indicated higher (more frequent) phubbing experiences (i.e., being phubbed).

#### Loneliness

Next, the short, 8-item form of the Revised UCLA Loneliness Scale [64] was used. Participants answered on a 4-point Likert scale ranging from 1 (never) to 4 (often). Example items included “*I am unhappy being so withdrawn*” and “*I feel left out*”. Cronbach’s alpha in the present study was 0.75. The scale was previously used in similar Romanian samples, suggesting its good psychometric properties [65].

#### Time spent on social media

Finally, participants were asked to self-reported their time spent on social media by answering the following questions: *How many hours do you spend, daily, using social media?*. The participants chose their answers on a scale ranging from 0 (none), 1 (less than 1 h a day), 2 (1 to 3 h daily), and 3 (more than 3 h a day).

A demographic scale assessed participants’ age, gender, and relational status.

### Overview of data analysis

The resulting data were analyzed using the 26v. of the SPSS program and Hayes’ Macro Process [66]. There were no missing data, as the items were set as required (i.e., mandatory) to complete the study. The initial assumptions assessment was performed by descriptive univariate analysis (see Table 1). Skewness and Kurtosis indicators were computed to assess the normality of the distributions. Internal consistency was assessed using the alpha Cronbach indicators. The correlations between the variables were examined, and then mediation analyses were performed using Macro Process (Model 4). The theoretical model was tested by estimating the 95% confidence interval (CI) for mediation effects with 5000 bootstrapped samples. Age, gender, relationship status, and the time spent online were introduced as covariates.

**Table 1:** Descriptive statistics for the main variables (N = 720)

Variable	M	SD	Min	Max	Skewness (SE)	Kurtosis (SE)
Psychological distress	60.77	18.69	21	105	− 0.15 (0.09)	− 0.60 (0.18)
Life satisfaction	23.77	6.37	5	35	-0.26 (0.09)	− 0.31 (0.18)
Perceived phubbing	64.99	18.69	22	110	0.04 (0.09)	-0.01 (0.18)
Loneliness	18.06	4.35	8	31	0.07 (0.09)	-0.37 (0.18)
Time on social media	2.37	0.67	0	3	-0.88 (0.09)	0.68 (0.18)

## Results

### The associations between the study’s variables

Descriptive statistics for the study variables are provided in Table 1. Correlation analyses suggested that phubbing was positively related to psychological distress (*r* = .37, *p* < .001; medium size effect; [67]) and loneliness (*r* = .30, *p* < .001; medium size effect; [67]), while the relation with life satisfaction was non-significant. Further, loneliness was negatively related to life satisfaction (*r* = − .34, *p* < .001; medium size effect; [67]) and positively related to psychological distress (*r* = .56, *p* < .001; large size effect; [67]). The results are presented in Table 2.

**Table 2:** Associations between the main variables (N = 720)

Variable	1	2	3	4
1. Psychological distress				
2. Life satisfaction	− 0.22**			
3. Perceived phubbing	0.37**	0.03		
4. Loneliness	0.56**	− 0.34**	0.30**	
5. Age	− 0.20**	0.09*	− 0.10*	− 0.19**

**p < .001; * *p* < .01

### The mediating effect of loneliness on the link between perceived phubbing and life satisfaction

**Fig. 2:**
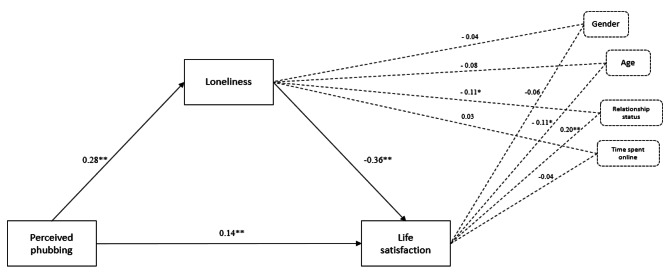
The mediating role of loneliness on the link between perceived phubbing and life satisfaction (controlling for age, gender, relational status, and time spent online), N = 720. Values represent standardized coefficients. **p < .001; * *p* < .05

The total effect of perceived phubbing on life satisfaction was not significant, *B* = 0.01, *SE* = 0.01, 95% CI [-0.00; 0.03]. The effect of perceived phubbing on loneliness was significant, *B* = 0.28, *SE* = 0.00, 95% CI [0.05; 0.08], *R* = .36, *R*^2^ = 0.13, *MSE* = 16.59, *F* (5; 714) = 21.70. In the model that included both predictors (*R* = .41, *R*^2^ = 0.17, *MSE* = 33.96, *F* (6; 713) = 24.67), loneliness was a significant predictor of life satisfaction, *B* = − 0.36, *SE* = 0.05, 95% CI [-0.64; -0.43]. The direct effect of perceived phubbing on life satisfaction (controlling for age, gender, relationship status, and time spent online) was significant, *B* = 0.05, *SE* = 0.01, 95% CI [0.02; 0.07], and so was the indirect effect, *B* = -0.03, *SE* = 0.00, 95% CI [-0.04; -0.02]. Thus, loneliness partially mediated the link between perceived phubbing and life satisfaction when controlling for age, gender, relationship status, and time spent online (see Fig. 2).

### The mediating effect of loneliness on the link between perceived phubbing and psychological distress

**Fig. 3:**
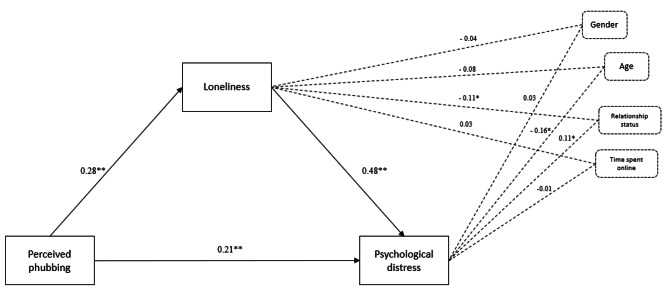
The mediating role of loneliness on the link between perceived phubbing and psychological distress (controlling for age, gender, relational status, and time spent online), N = 720. Values represent standardized coefficients. **p < .001; * *p* < .05

The total effect of perceived phubbing on psychological distress was significant, *B* = 0.35, *SE* = 0.03, 95% CI [0.29; 0.42]. The effect of perceived phubbing on loneliness was significant, *B* = 0.28, *SE* = 0.00, 95% CI [0.05; 0.08], *R* = .36, *R*^2^ = 0.13, *MSE* = 16.59, *F* (5; 714) = 21.70. In the model that included both predictors (*R* = .61, *R*^2^ = 0.37, *MSE* = 219.96, *F* (6; 713) = 71.58), loneliness was a significant predictor of psychological distress, *B* = 0.48, *SE* = 0.13, 95% CI [1.82; 2.35]. The direct effect of perceived phubbing on psychological distress (controlling for age, gender, relationship status, and time spent online) was significant, *B* = 0.21, *SE* = 0.03, 95% CI [0.15; 0.28], and so was the indirect effect, *B* = 0.13, *SE* = 0.02, 95% CI [0.10; 0.18]. Thus, loneliness partially mediated the link between perceived phubbing and psychological distress when controlling for age, gender, relationship status, and time spent online (see Fig. 3).

## Discussion

The present study examined the relationships between perceived phubbing, life satisfaction, and psychological distress and the mediating role of loneliness in the relationship between phubbing with life satisfaction and psychological distress. One of the strengths of this study results from the investigated sample, i.e., adults, a rather understudied population regarding phubbing, since most studies focused on teenagers [27].

The assumptions made stated that loneliness would mediate the link between (a) phubbing and life satisfaction and (2) phubbing and psychological distress. In these analyses, age, gender, relationship status, and time spent online were used as covariates, given the extended literature underlying their potential influence on all the study variables [41, 43, 45]. The findings sustained these hypotheses. In line with previous literature [24], a significant relationship was found between phubbing, life satisfaction, and psychological distress. Thus, among the detrimental factors associated with phubbing may not only its potential to increase psychological distress but also the possibility to decrease life satisfaction; however, these implications need to be addressed in future longitudinal studies, which allow the examination of causality.

From a theoretical point of view, these results align with the stress-coping model [52] that the assumptions were based on. According to this model, phubbing might act as a significant stressor that would further have a significant influence on phubbees’ psychological distress and life satisfaction, since perceived phubbing leads to loneliness, which challenges one’s coping resources [53, 55]. More specifically, the present findings indicated that perceived phubbing was significantly associated with loneliness while also showing an indirect relationship with life satisfaction. This aligns with the stress-coping model’s first step of appraisal, where individuals perceive a situation (in this case, phubbing) as a potential stressor [68]. Next, the stress-coping model [52] suggests that appraisals of stressors may lead to specific emotional and cognitive responses; in the present research, loneliness was a significant mediator in the relationship between perceived phubbing and both psychological distress and life satisfaction. This suggests that the experience of being ignored due to phone usage triggers feelings of loneliness, which might further contribute to increased psychological distress and decreased life satisfaction.

Next, the stress-coping model also highlights the role of coping strategies in managing stress [69]. In the present study, the coping strategies individuals employ to deal with the stress generated by perceived phubbing might be influenced by the loneliness it generates. This might lead to maladaptive coping strategies that can exacerbate psychological distress and hinder effective management of such situations. Finally, the stress-coping model posits that the interplay between stressor appraisals, emotional responses, and coping strategies influences psychological outcomes. The current results suggested that loneliness significantly mediated the relationship between perceived phubbing, psychological distress, and life satisfaction, indicating that the experience of phubbing triggers a cascade of responses that ultimately impact mental well-being.

Thus, the present research highlighted the detrimental effects of feeling ignored and rejected (perceived phubbing), which are significantly associated with loneliness, further leading to higher depression, anxiety, and stress, and lower life satisfaction, a pattern previously highlighted in studies focused on interpersonal relationships [70, 71]. Nevertheless, these theoretical implications need further validation in future studies that would add to the related literature. Furthermore, these results highlight the need to understand better the predictors and outcomes of phubbing behaviors (i.e., from the phubbees’ perspective) to shape effective interventions. Prevention and intervention programs might focus and detail (a) the importance of uninterrupted, focused attention toward others during a one-on-one, face-to-face interaction; (b) the benefits of paying attention to the person in front of us to comprehend better and – subsequently – to react to the person we are communicating/interacting with. In both prevention and intervention programs and strategies, we might also focus on and detail the adverse outcomes of phubbing behavior (e.g., loneliness, depression, anxiety, and stress symptoms) to clearly and specifically highlight the consequences of paying attention to smartphones, instead of people [72].

Previous studies based on phubbing experiences (i.e., agents) suggested that phubbing may be significantly and negatively linked to life satisfaction [14]. The present results, however, add to the related literature and suggest that this association (though indirect, through loneliness), is also significant in the case of phubbees. Furthermore, the current results also align with previous findings suggesting that suggested that partner phubbing has a significant indirect impact on life satisfaction [15]. Though the focus in the present research was not on partner phubbing (see the instructions participants received), the current findings add to the literature in this regard, shaping a more general view of the indirect effect of phubbing on life satisfaction. Also, the present findings align with previous literature suggesting that phubbing experiences negatively impact feelings of connectedness [19, 20], which are significantly and negatively related to loneliness [29].

The present findings did not highlight a significant direct relation between perceived phubbing and life satisfaction. This result aligns with previous similar findings, which suggested a rather indirect (mediated) relationship between these variables [27]. This specific result highlights the need to examine the various possible mediators between perceived phubbing and various outcomes since the impact of phubbing on life satisfaction might not be straightforward due to various factors, such as individual differences. Furthermore, the present results may contribute to the advancement of existing theoretical frameworks by highlighting the role of mediating variables in explaining the complex relationship between technology-related behaviors (like phubbing) and psychological outcomes (like life satisfaction and psychological distress).

There are several limitations to be considered when interpreting the current results. First, self-reported measures were used, which may have increased participants’ desirable answers. Future studies might benefit from using experimental approaches, which might help understand better the investigated relationships. For example, instead of measuring the perceived phubbing using self-reported scales, experimental procedures might be employed to observe in natural contexts this phenomenon and analyze its psychological implications. This way, loneliness might be explored as a temporary state potentially caused by perceived phubbing. Furthermore, this would imply using longitudinal measurements instead of cross-sectional data, increasing the findings’ generalizability and generating the possibility of drawing causal relations between the primary variables in question. Similarly, Internet use might be approached more objectively in future studies (i.e., more specific measures instead of self-reported time spent online).

Next, though participants’ gender, age, relationship status, and time spent online were controlled, future studies might benefit from integrating these variables into the research models, using more extended and balanced samples. Also, future studies might benefit from examining the social displacement hypothesis in relation to phubbing experiences, given the interesting results presented by Verduyn et al. [73] on daily smartphone and face-to-face communication. Additionally, future studies might benefit from examining the way loneliness affects perceived phubbing: it is possible that participants who experience high levels of loneliness might perceive phubbing differently (i.e., more intense) than those with lower perceived loneliness. Also, future studies might benefit from examining the proposed relations in more gender-balanced samples, to examine the potential differences between male and female participants regarding perceived phubbing experiences and their ramifications on psychological distress and life satisfaction [74].

Finally, various other factors might account for significant variations regarding perceived phubbing and loneliness (e.g., negative self-evaluation, feelings of belongingness; [18, 24]), life satisfaction (e.g., religion and spirituality [75],; compassion [76],), and psychological distress (e.g., growth mindsets [77],). Future studies might explore these variables as moderators in the proposed research model to better understand the complex psychological outcomes when discussing the overlap between the digital and the non-digital world.

## Conclusion

The present findings might add important insights regarding the significant role of perceived phubbing when discussing psychological distress and life satisfaction, underlining the need to address further the (mis)use of digital devices (e.g., smartphones) within interpersonal relationships. The present results also highlight the role of loneliness as a mechanism linking phubbing with life satisfaction and psychological distress. Further studies could explore other mechanisms, as well as the protective factors that may moderate the relation between phubbing and mental health indicators.

### Electronic supplementary material

Below is the link to the electronic supplementary material.

Supplementary Material 1

## Data Availability

The data supporting this study’s findings are available at: Alexandra Maftei, & Cornelia Mairean. (2023). Data set for “Perceived phubbing, life satisfaction, and psychological distress: The mediating role of loneliness” [Data set]. Zenodo. 10.5281/zenodo.7614459.

## References

[1] Datareportal.com. [cited 2023 Aug 21]. Available from: https://datareportal.com/reports/digital-2021-global-digital-overview

[2] Lozano-Blasco R, Robres AQ, Sánchez AS (2022). Internet addiction in young adults: a meta-analysis and systematic review. Comput Hum Behav.

[3] Jonsson U, Linton SJ, Ybrandt H, Ringborg A, Leander L, Moberg K (2023). Internet-delivered psychological treatment as an add-on to treatment as usual for common mental disorders: a systematic review with meta-analysis of randomized trials. J Affect Disord [Internet].

[4] Dwyer RJ, Kushlev K, Dunn EW (2018). Smartphone use undermines enjoyment of face-to-face social interactions. J Exp Soc Psychol [Internet].

[5] Stop Phubbing. (2019). *Definition and disturbing phubbing stats*. Retrieved from https://web.archive.org/web/20120901204042/http://stopphubbing.com/

[6] Balta S, Emirtekin E, Kircaburun K, Griffiths MD. Neuroticism, trait fear of missing out, and phubbing: The mediating role of state fear of missing out and problematic Instagram use. Int J Ment Health Addict [Internet]. 2020;18(3):628–39. 10.1007/s11469-018-9959-8

[7] Nazir T, Pişkin M, Phubbing (2016). A technological invasion which connected the world but disconnected humans. Int J Ind Psychol.

[8] Çikrikci Ö, Griffiths MD, Erzen E (2022). Testing the Mediating Role of Phubbing in the Relationship between the big five personality traits and satisfaction with life. Int J Ment Health Addiction.

[9] Kadylak T (2020). An investigation of perceived family phubbing expectancy violations and well-being among U.S. older adults. Mob Media Commun [Internet].

[10] Kadylak T, Makki TW, Francis J, Cotten SR, Rikard RV, Sah YJ (2018). Disrupted copresence: older adults’ views on mobile phone use during face-to-face interactions. Mob Media Commun [Internet].

[11] Kadylak T (2020). An investigation of perceived family phubbing expectancy violations and well-being among U.S. older adults. Mob Media Communication.

[12] Grieve R, March E (2021). Just checking’: vulnerable and grandiose narcissism subtypes as predictors of phubbing. Mob Media Communication.

[13] Al-Saggaf Y, O’Donnell SB, Phubbing (2019). Perceptions, reasons behind, predictors, and impacts. Hum Behav Emerg Technol.

[14] Parmaksiz İ (2021). Predictive effect of phubbing and life satisfaction on depression symptoms in adults. Bağımlılık derg.

[15] Roberts JA, David ME (2016). My life has become a major distraction from my cell phone: Partner phubbing and relationship satisfaction among romantic partners. Comput Hum Behav.

[16] McDaniel BT, Coyne SM (2016). Technoference: the interference of technology in couple relationships and implications for women’s personal and relational well-being. Psychol Pop Media Cult.

[17] Vanden Abeele MMP, Postma-Nilsenova M (2018). More than just gaze: an experimental vignette study examining how phone-gazing and newspaper-gazing and phubbing-while-speaking and phubbing while-listening compare in their effect on affiliation. Communication Res Rep.

[18] Chotpitayasunondh V, Douglas KM (2018). The effects of phubbing on social interaction. J Appl Soc Psychol [Internet].

[19] Misra S, Cheng L, Genevie J, Yuan M (2014). The iPhone effect: the quality of in-person social interactions in the presence of mobile devices. Environ Behav.

[20] Przybylski AK, Weinstein N (2013). Can you connect with me now? How the presence of mobile communication technology influences face-to-face conversation quality. J Soc Pers Relat [Internet].

[21] Al-Saggaf Y (2022). Social norms. SpringerBriefs in psychology.

[22] Bitar Z, Akel M, Salameh P, Obeid S, Hallit S. Phubbing among lebanese young adults: scale validation and association with mental health (depression, anxiety, and stress). Curr Psychol [Internet]. 2022;1–12. 10.1007/s12144-022-03104-z PMC9039595 35496363

[23] Chatterjee S (2020). Antecedents of phubbing: from technological and psychological perspectives. J Syst Inf Technol [Internet].

[24] Ergün N, Göksu İ, Sakız H (2020). Effects of phubbing: Relationships with psychodemographic variables. Psychol Rep [Internet].

[25] Zhang Y, Ding Q, Wang Z (2021). Why parental phubbing is at risk for adolescent mobile phone addiction: a serial mediating model. Child Youth Serv Rev [Internet].

[26] Chu X, Ji S, Wang X, Yu J, Chen Y, Lei L. Phubbing while phoning: an instrumental multiple-case study of college students’ smartphone use. Front Psychol. 2021;12.

[27] Liu K, Wang J, Wei X, Lei L (2023). Parental modeling and normative influence in shaping teenagers’ phubbing: an exploratory study. Youth Soc [Internet].

[28] Peplau LA, Perlman D (1982). Perspectives on loneliness. Loneliness: a sourcebook of current theory, research and therapy, 1–18.

[29] Cacioppo S, Grippo AJ, London S, Goossens L, Cacioppo JT (2015). Loneliness: clinical import and interventions. Perspect Psychol Sci [Internet].

[30] Moretta T, Buodo G (2020). Problematic internet use and loneliness: how complex is the relationship? A short literature review. Curr Addict Rep [Internet].

[31] Bucher A, Neubauer AB, Voss A, Oetzbach C (2019). Together is better: higher committed relationships increase life satisfaction and reduce loneliness. J Happiness Stud.

[32] McGinty EE, Presskreischer R, Han H, Barry CL (2020). Psychological distress and loneliness reported by US adults in 2018 and April 2020. JAMA.

[33] Latikka R, Koivula A, Oksa R, Savela N, Oksanen A (2022). Loneliness and psychological distress before and during the COVID-19 pandemic: Relationships with social media identity bubbles. Soc Sci Med.

[34] Wu X, Zhang L, Yang R, Zhu T, Xiang M, Wu G (2022). Parents can’t see me, can peers see me? Parental phubbing and adolescents’ peer alienation via the mediating role of parental rejection. Child Abuse Negl.

[35] Clear SJ, Zimmer-Gembeck MJ, Duffy AL, Barber BL (2020). Internalizing symptoms and loneliness: direct effects of mindfulness and protection against the negative effects of peer victimization and exclusion. Int J Behav Dev.

[36] Xiao B, Bullock A, Liu J, Coplan R (2021). Unsociability, peer rejection, and loneliness in Chinese early adolescents: testing a cross-lagged model. J Early Adolesc.

[37] Xu X-P, Liu Q-Q, Li Z-H, Yang W-X (2022). The mediating role of loneliness and the moderating role of gender between peer phubbing and adolescent mobile social media addiction. Int J Environ Res Public Health.

[38] Chen I-H, Pakpour AH, Leung H, Potenza MN, Su J-A, Lin C-Y (2020). Comparing generalized and specific problematic smartphone/internet use: longitudinal relationships between smartphone application-based addiction and social media addiction and psychological distress. J Behav Addict.

[39] Błachnio A, Przepiorka A, Pantic I (2016). Association between Facebook addiction, self-esteem and life satisfaction: a cross-sectional study. Comput Hum Behav.

[40] Ivanova A, Gorbaniuk O, Błachnio A, Przepiórka A, Mraka N, Polishchuk V, Gorbaniuk J (2020). Mobile phone addiction, phubbing, and Depression among Men and Women: a Moderated Mediation Analysis. Psychiatr Q.

[41] Jung Y, Lee YK, Hahn S. Age and gender differences in loneliness during the COVID-19: Analyses on large cross-sectional surveys and emotion diaries [Internet]. PsyArXiv. 2022.

[42] Wickens CM, McDonald AJ, Elton-Marshall T, Wells S, Nigatu YT, Jankowicz D (2021). Loneliness in the COVID-19 pandemic: Associations with age, gender and their interaction. J Psychiatr Res [Internet].

[43] Barrantes R, Vargas E. Different paths and same destinations? An analysis of the convergence in internet usage patterns between different age groups. Electron J Inf Syst Dev Ctries [Internet]. 2019;85(6).

[44] Adamczyk K, Segrin C (2015). Direct and Indirect Effects of young adults’ relationship status on life satisfaction through loneliness and perceived social support. Physiol Belgica.

[45] Böger A, Huxhold O (2020). The changing relationship between partnership status and loneliness: Effects related to aging and historical time. J Gerontol B Psychol Sci Soc Sci [Internet].

[46] Dush CMK, Amato PR (2005). Consequences of relationship status and quality for subjective well-being. J Soc Pers Relat [Internet].

[47] Leach LS, Butterworth P, Olesen SC, Mackinnon A (2013). Relationship quality and levels of depression and anxiety in a large population-based survey. Soc Psychiatry Psychiatr Epidemiol.

[48] Stepanikova I, Nie NH, He X (2010). Time on the internet at home, loneliness, and life satisfaction: evidence from panel time-diary data. Comput Hum Behav.

[49] Costa RM, Patrão I, Machado M (2019). Problematic internet use and feelings of loneliness. Int J Psychiatry Clin Pract.

[50] Davey A, Davey S, Raghav S, Singh J, Singh N, Blachnio A (2018). Predictors and consequences of phubbing among adolescents and youth in India: an impact evaluation study. J Family Community Med.

[51] Tomczyk Ł, Lizde ES (2022). Nomophobia and Phubbing: Wellbeing and new media education in the family among adolescents in Bosnia and Herzegovina. Child Youth Serv Rev.

[52] Lazarus RS, Folkman S (1984). Stress, Appraisal, and coping.

[53] Pearlin LI, Schieman S, Fazio EM, Meersman SC (2005). Stress, health, and the life course: some conceptual perspectives. J Health Soc Behav.

[54] Biggs A, Brough P, Drummond S. Lazarus and Folkman’s psychological stress and coping theory. The handbook of stress and health: A guide to research and practice. 2017;349–64.

[55] Zhang X, Dong S (2022). The relationships between social support and loneliness: a meta-analysis and review. Acta Psychol (Amst).

[56] Carr D, Mooney H. Bereavement in later life. Handbook of aging and the social sciences. Academic Press; 2021. 239–54.

[57] Faul F, Erdfelder E, Lang A-G, Buchner A (2007). G*Power 3: a flexible statistical power analysis program for the social, behavioral, and biomedical sciences. Behav Res Methods.

[58] Schoemann AM, Boulton AJ, Short SD (2017). Determining power and sample size for simple and complex mediation models. Soc Psychol Personal Sci [Internet].

[59] Hambleton RK, Li S. Translation and adaptation issues and methods for Educational and Psychological tests. In: Frisby CCLR, editor. Comprehensive handbook of multicultural school psychology. John Wiley & Sons, Inc; 2005. pp. 881–903.

[60] Lovibond PF, Lovibond SH (1995). The structure of negative emotional states: comparison of the Depression anxiety stress scales (DASS) with the Beck Depression and anxiety inventories. Behav Res Ther [Internet].

[61] Foti FL, Karner-Huţuleac A, Maftei A (2023). The value of motherhood and psychological distress among infertile women: the mediating role of coping strategies. Front Public Health [Internet].

[62] Măirean C, Zancu SA, Diaconu-Gherasim LR, Brumariu LE (2023). Mental health among young adults during the COVID-19 pandemic: a two-wave longitudinal investigation. J Psychol.

[63] Diener E. Larsen, Griffin. The satisfaction with life scale. J Pers Assess; 1985. p. 71–5. 10.1207/s15327752jpa4901_13 16367493

[64] Hays RD, DiMatteo MR. A short-form measure of loneliness. J Pers Assess. 1987 Spring;51(1):69–81. 10.1207/s15327752jpa5101_6. PMID: 3572711. 10.1207/s15327752jpa5101_6 3572711

[65] Pop LM, Iorga M, Iurcov R (2022). Body-esteem, self-esteem and loneliness among social media young users. Int J Environ Res Public Health.

[66] Hayes AF. Introduction to mediation, moderation, and conditional process analysis: a regression-based approach. Guilford Press; 2013.

[67] Cohen J (2013). Statistical power analysis for the behavioral sciences [Internet].

[68] Kim M-S, Duda JL (2003). The coping process: cognitive appraisals of stress, coping strategies, and coping effectiveness. Sport Psychol.

[69] El-Ghoroury NH, Galper DI, Sawaqdeh A, Bufka LF (2012). Stress, coping, and barriers to wellness among psychology graduate students. Train Educ Prof Psychol [Internet].

[70] Rotenberg KJ. Peer rejection and loneliness. The Encyclopedia of Child and Adolescent Development. 2019;1–8.

[71] Wang X, Gao L, Yang J, Zhao F, Wang P (2020). Parental phubbing and adolescents’ depressive symptoms: self-esteem and perceived social support as moderators. J Youth Adolesc.

[72] Han JH, Park S-J, Kim Y (2022). Phubbing as a millennials’ new addiction and relating factors among nursing students. Psychiatry Investig.

[73] Verduyn P, Schulte-Strathaus JCC, Kross E, Hülsheger UR (2021). When do smartphones displace face-to-face interactions and what to do about it?. Comput Hum Behav.

[74] Xie X, Chen W, Zhu X, He D (2019). Parents’ phubbing increases adolescents’ Mobile phone addiction: roles of parent-child attachment, deviant peers, and gender. Child Youth Serv Rev.

[75] Yaden DB, Batz-Barbarich CL, Ng V, Vaziri H, Gladstone JN, Pawelski JO (2022). A meta-analysis of religion/spirituality and life satisfaction. J Happiness Stud [Internet].

[76] Gu X, Luo W, Zhao X, Chen Y, Zheng Y, Zhou J (2022). The effects of loving-kindness and compassion meditation on life satisfaction: a systematic review and meta-analysis. Appl Psychol Health Well Being [Internet].

[77] Burnette JL, Knouse LE, Vavra DT, O’Boyle E, Brooks MA (2020). Growth mindsets and psychological distress: a meta-analysis. Clin Psychol Rev [Internet].

