# Supplementary material for: Put your phone down! Perceived phubbing, life satisfaction, and psychological distress: the mediating role of loneliness
Source: BMC Psychol. 2023 Oct 12;11:332. doi: 10.1186/s40359-023-01359-0 (PMC10571372; doi:10.1186/s40359-023-01359-0)
Supplement: Supplementary file 1 — Supplementary Material 1 [file 40359_2023_1359_MOESM1_ESM.docx]

**Appendix**

Measures

a) **The Depression, Anxiety, and Stress Scale** (DASS-21; Lovibond & Lovibond, 1995) – the Romanian version (Foti et al., 2023; Măirean et al., 2023) was used to measure participants’ depression, anxiety, and stress symptoms, i.e., psychological distress. We used **a total score** of the scale, and not its dimensions.

- **Original scale**: In the original study (Lovibond & Lovibond, 1995), Cronbach’s alpha-s were the following: α = 0.91 for depression, α = 0.81 for anxiety, and α = 0.89 for stress.
- **The adapted scale** used in this study = the Romanian version of the DASS-21 scale, previously used by Foti et al. (2023) and Măirean et al. (2023).
- **In the study conducted by Foti et al**. (2023) – the authors used the total score of the scale, as in the present study, with Cronbach’s alpha-s of: α = 0.89 for depression, α = 0.90 for anxiety, and α = 0.89 for stress.
- **In the study conducted by Măirean et al., 2023**, Cronbach’s alpha was 0.95
- **In the present study,** Cronbach’s α for the overall scale was 0.95.
- The confirmatory factorial analysis sustains the model with the three factors: χ^2^(132) = 379.33, *p* < .001; CFI = .97; RMSEA = .05, 95% CI [.04 - .06].

**b) The Life satisfaction scale:** The Satisfaction with Life Scale developed by Diener et al. (1985) was used. Following the original guidelines, from the authors of the scale, the scoring was kept continuous (summing up scores on each item = total score of the scale).

- **Original scale**: In the original study (Diener et al., 1985), Cronbach’s alpha was 0.87.
- **The adapted scale** used in this study = the Romanian version of the scale, previously used by [Maftei and Lazarescu (2022)](https://doi.org/10.3389/fpsyg.2022.915524)
- **In the study conducted by** [**Maftei and Lazarescu** (2022](https://doi.org/10.3389/fpsyg.2022.915524)), Cronbach’s α was 0.86
- **In the present study,** Cronbach’s α was 0.88.
- CFA is not needed, since this is a unidimensional scale.

**c) The Perceived Phubbing Scale**: Phubbing Experiences were measured using the 22-item Generic Scale of Being Phubbed developed by Chotpitayasunondh et al. (2018). The scale comprises three factors, i.e., ***Perceived Norms***, ***Feeling Ignored***, and ***Interpersonal Conflict***. We used **a total score** of the scale, and not its dimensions. In the present study, Cronbach’s α for the overall score was 0.95.

- **Original scale**: In the original study (Chotpitayasunondh et al. 2018), Cronbach’s alpha-s were the following: Perceived Norms (α = 0.92), Feeling Ignored (α = 0.94), and Interpersonal Conflict (α = 0.90).
- The present study = the translated version of the scale. **In the present study,** Cronbach’s α-s were: ***Perceived Norms*** (α = 0.92), ***Feeling Ignored*** (α = 0.95), and ***Interpersonal Conflict*** (α = 0.91)
- The confirmatory factorial analysis sustains the model with the three factors: χ^2^(169) = 512.80, *p* < .001; CFI = .97; RMSEA = .05, 95% CI [.04 - .06].

**d) The Loneliness scale** - the Revised UCLA Loneliness Scale (Hays, 1987)

- **The adapted scale** used in this study = the Romanian version of the scale, previously used by [Maftei and Mairean (2023)](https://doi.org/10.1080/10508422.2023.2230505)
- **In the study conducted by** [Maftei and Mairean (2023)](https://doi.org/10.1080/10508422.2023.2230505), Cronbach’s alpha was 0.75.
- The scale was previously used in similar Romanian samples (e.g., [Pop et al., 2022](https://doi.org/10.3390/ijerph19095064)) and indicated reliable psychometric properties.
- **In the present study**, Cronbach’s alpha was 0.75.
- CFA is not needed, since this is a unidimensional scale.
